# The Treatment of Corneal Ulcers by the General Practitioners

**Published:** 1905-12

**Authors:** Clarence Porter Jones

**Affiliations:** Newport News, Va., Member Staff Newport News General Hospital; Consulting Oculist and Aurist to National Soldiers’ Home Hospital, Dixie Hospital Hampton, Va., The Normal School Hospital, Etc.


ATLANTA

### Journal-Record of Medicine

Successor to Atlanta Medical and Surgical Journal, Established 1855, and Southern Medical Record, Established 1870.

OWNED BY THE ATLANTA MEDICAL JOURNAL CO.

**Published Monthly.**

VOL. VII. DECEMBER, 1905. No. 9.

BERNARD WOLFF, M.D., EDITOR, Nos. 319–20 Prudential.

M. B. HUTCHINS, M.D., BUSINESS MANAGER, 1007–1008 Century Bldg.

E. G. BALLENGER, M.D., ASSOCIATE EDITOR AND ASSISTANT MANAGER.
J. N. LECONTE, M.D., FOREIGN CORRESPONDENT.

### THE TREATMENT OF CORNEAL ULCERS BY THE GENERAL PRACTITIONERS.

BY CLARENCE PORTER JONES, M.D., NEWPORT NEWS, VA.,

Member Staff Newport News General Hospital; Consulting Oculist and Aurist to National Soldiers' Home Hospital, Dixie Hospital Hampton, Va., The Normal School Hospital, Etc.

Having recently treated twenty-two cases of ulcer of the cornea, all successfully, and to have had the misfortune to have three cases coming from the country districts, each with a blind eye as a sequel to corneal ulcer, I feel constrained to say a few words about the treatment of these cases by the general practitioner.

It is not the purpose of this little paper to, in any sense, discourage the practitioner in sending his cases to a competent oculist, which is his highly important duty. There is no disease of the human body which requires more skill and judgment in treatment than this malady. But there often occurs cases of ulcer of the cornea in the practice of a physician, especially in the country, which for financial or other reasons can not go to consult an oculist. These cases and only these should the physician undertake the responsibility of treating.

The cornea is a non-vascular structure, nourished by lymph osmosis from the lymph-spaces in the conjunctiva and sclera, which lie in juxtaposition to its own. Thus we see it is feebly resistant to infection.

Upon careful inquiry we find a vast majority of ulcers are due to an injury or abrasion which has become infected, either at the time of injury or subsequently thereto. It is the method of cure of this ulcer we discuss.

Destruction of the corneal substance is the essential feature of all forms of suppurative keratitis, leaving an opaque cicatrix at best on healing; or by perforation involve the whole eyeball resulting in phthisis bulbi and total loss of vision.

As before stated, an ulcer begins with a focus of infection, superficially situated, with ragged edges of a yellow color, being surrounded by a zone of infiltration. Its sides and bottom are covered by a detritus of a pultaceous appearance. Conjunctival congestion, photophobia, lachrymation and pain, varying greatly with individual cases, is present.

Treatment should be both preventive and therapeutic. While I do not claim anything new in the line of treatment I follow, yet I think I am justified in presenting it, as it has proven itself to be the very best in my hands after hard and persistent efforts.

Every injury of the cornea should be assumed to be infected. The eye should be promptly irrigated with a saturated solution of boric acid, or bichloride of mercury solution one to four thousand, and kept as nearly aseptic as is possible to so do by use of a bichloride and salt ointment containing bichloride of mercury one-fifth grain, and sodium chloride one grain to vaseline one ounce. If pain is a factor, I add five grains of cocain, allowing the druggist to use a small quantity of liquid albolene to better enable the cocain to dissolve. If the injury is as much as twelve hours old and the proper antiseptic precautions have not been taken, I touch the wound with tincture of iodine applied by a few shreds of absorbent cotton wound around a small probe or smooth wooden toothpick. All applications to the cornea being painful a previous instillation of a six per cent. solution of cocain should be made.

When the ulcer has declared itself my first procedure is to curette thoroughly with small size Meyhoefer's corneal curette, then touch with the tincture of iodine. If after twenty-four hours there is no marked improvement, cauterization, either with carbolic acid after the same manner as the application of iodine, or the actual cautery, should be thoroughly done; for the latter, the suitable instrument being Gruening's cautery probe. These two instruments are very inexpensive and should be in the office of every country doctor. Atropine sulphate, one per cent., should be instilled every four hours for the first day, thence twice a day. Hot applications, boric acid, a teaspoonful to a pint of water, as hot as can be borne, every hour, bathing eye for ten minutes at a time, are very beneficial.

A shade or smoked glass may be worn to shield the eye from light.

The cauterization may be repeated in two or three days, if necessary; often one application suffices. In bad cases, and especially highly nervous patients, rest in bed in a dark room, and nutritious diet is imperative. Pain is usually relieved by the application of the vaseline preparation with cocain above referred to. This can be augmented by codeine by the mouth, or hypodermically. The curette is highly important, as it removes tissue which would otherwise slough away, were it not used; therefore causing no more scar than would otherwise result. Then, too, by its removal of the detritus, the iodine and other antiseptics are enabled to have a clear field of action.

The line of treatment here laid down caused prompt results in the twenty-two cases mentioned at the beginning of this paper. Each case was preceded by an abrasion, or injury, scarcely any two from the same source.

I will report three cases seen by me from five to twenty weeks after the initial abrasion, which had not had treatment:

CASE I. Mary S., age thirteen; was hit in the eye by a briar twig while picking berries. Pain and discomfort being so slight, little attention was paid to it for the time being. Four days later she saw her family physician, who lives six miles from her home. She had a well defined ulcer a little larger than a pin head, on the upper outer margin of the cornea. The doctor told her father this, and also the danger there was of the loss of the eye, and insisted that an oculist be seen as quickly as possible. For financial reasons, this was impossible (the nearest oculist being fifty miles away). Whereupon the doctor prescribed a boric acid wash, and told her to report back in a few days.

Eight days later the cornea had perforated, the lens presenting panophthalmitis, with destruction of sight. About four weeks later, while spending my vacation in her vicinity, I enucleated the eye on account of sympathetic trouble in its fellow—it being permanently damaged.

CASE II. Arthur B., a colored youth; was struck in the eye last Christmas by the seeds of a torpedo (not a burn). Pain was slight. Next a small oval abrasion was seen in the center of the cornea. Two days later he consulted his family physician to obtain relief from pain. At this time an oval superficial ulcer, the size of a grain of wheat, covering the site of injury was present. The doctor prescribed a poultice of meal bran to the eye. A virulent suppurative keratitis followed, which destroyed over half the thickness of the cornea, though not perforating it. Four months later this physician sent the case to me. An enormous staphyloma had developed, the eye very painful, and was totally blind. There was photophobia, excessive lachrymation, and some congestion in the fellow eye. I promptly enucleated the destroyed eye.

CASE III. Ephriam W., age thirty years; was struck in the eye by a bundle of wheat while feeding a threshing machine. There was sharp pain for a couple of hours, then comparative relief. About three days later his physician diagnosed a creeping ulcer covering the lower inner quadrant of the cornea. He was promptly told of his danger, and advised to go at once to a neighboring oculist; he could not do so on account of sickness in his family. His doctor gave him a solution of bichloride of mercury wash, and a solution of eserine to instill.

The case went from bad to worst, speedily developing hypopyon, plastic iritis, occluded pupil, blindness, and later atrophy of the ball. Two months later I removed the eye to relieve excessive pain.

A sad picture this! Here are three eyes lost from want of proper, though simple, treatment. The chances are greatly in favor that each doctor could have saved his case.

In each case an oculist was out of the question; then it was the duty of the physician to have done the best in his power. In such cases should be followed the old, though simple, treatment as mentioned here. The chances are that good results can be accomplished. Cocaine and atropine solutions should be made up in a bichloride of mercury, one to three thousand vehicle, to which may be added one grain of sodium chloride. If some antiseptic vehicle is not used, they will deteriorate and give uncertain results.

My observation of the treatment of corneal ulcers in the great clinics of America and Europe will bear out all assertions herein made.

In conclusion, I will state:

I. All abrasions of the cornea, however slight, should be assumed to be infected and treated antiseptically, using iodine if the injury is twelve hours old.

II. That it is the imperative duty of a physician to send his corneal ulcers, on account of the danger of destruction to eyesight, to an oculist of merit as quickly as possible.

III. That should there be any reason which forbids this, it is his urgent duty to treat the case as vigorously as if it were a case of ophthalmia neonatorum.

IV. That in emergency the physician should use the corneal curette freely and feel that the sin of omission is fourfold to the sin of commission.

118 Thirty-second street.

